# Supplementary material for: Online training and pruning of multi-wavelength photonic neural networks
Source: Nanophotonics. 2025 Nov 10;14(27):5035–46. doi: 10.1515/nanoph-2025-0296 (PMC12717894; doi:10.1515/nanoph-2025-0296)
Supplement: Supplementary file 1 — Supplementary Material Details [file j_nanoph-2025-0296_suppl_001.pdf]

# Supplementary Information for "Online Training and Pruning of Multi-Wavelength Photonic Neural Networks"

Jiawei Zhang[1,4*], Weipeng Zhang[1,4], Tengji Xu[2,4], Lei Xu[1], Eli A. Doris[1], Bhavin J. Shastri[3], Chaoran Huang[2], and Paul R. Prucnal[1]

[1] *Department of Electrical and Computer Engineering, Princeton University, Princeton, 08544, New Jersey, USA*
[2] *Department of Electronic Engineering, The Chinese University of Hong Kong, Shatin, Hong Kong SAR, China*
[3] *Department of Physics, Engineering Physics and Astronomy, Queen's University, Kingston, K7L 3N6, Ontario, Canada*
[4] *The authors contribute equally to this paper.*
[*] *Corresponding author: jiawei.zhang@princeton.edu*

## Table of Contents

1 Look-up Table Modeling Non-idealities

2 Experimental Setup with Photonic Packaging

3 Demonstration on Iris Dataset
- 3.1 Definition of Loss function
- 3.2 Data Generation and Modulation

4 Simulations on Large-scale NNs
- 4.1 Datasets and NN setup
- 4.2 Modeling and Procedure
- 4.3 Energy Saving Details via Pruning

5 System Latency Analysis

6 Comparison with other works

# 1 Look-up Table Modeling Non-idealities

In the MRR-based PNNs, the development of a static look-up table (LUT) accounting for all the non-idealities can be fairly complicated. In an MRR weight bank using thermo-optic tuning to counteract chip-to-chip variations, a simplified physical model presented in [1] gives:

$$\mathbf{w}_{ij} \equiv \Lambda(\lambda_{ij}; \lambda_{ij,0}, \Delta\lambda_{ij}) \cdot P_{j,\text{opt}}/NP_0, \tag{S1}$$

$$\Lambda(\lambda_{ij}; \lambda_{ij,0}, \Delta\lambda_{ij}) = 1 - (1 + (\lambda_{ij} - \lambda_{ij,0})^2/\Delta\lambda_{ij}^2)^{-1}, \tag{S2}$$

where $\mathbf{w}_{ij}$ is the weight to be executed on the $i, j$th MRR, $\Lambda(\lambda_{ij}; \lambda_{ij,0}, \Delta\lambda_{ij})$ is the Lorentzian transfer function; $\lambda_{ij}(\lambda_{ij,0})$ is the resonance wavelength of the $i, j$th MRR with (without) applied heat, respectively; $\Delta\lambda_{ij}$ is the half-width half-maximum of Lorentzian transfer function; $P_{j,\text{opt}}$ is the input optical power at the $j$th laser wavelength; $P_0$ is a normalization constant. The wavelength offset compensating for fabrication variations ($\sigma_{ij}$), can be expressed as

$$\sigma_{ij} = \lambda_{ij} - \lambda_{ij,0} = \lambda_{ij,0}\frac{\beta_{\text{eff},ij}\Delta T_{ij}}{n_{\text{eff}}\left(\lambda_{ij,0}\right)}, \tag{S3}$$

where $\beta_{\text{eff},ij}$ is the thermo-optic coefficient, $n_{\text{eff}}\left(\lambda_{ij,0}\right)$ denotes the effective index, $\Delta T_{ij}$ is the temperature offset, which is given by Joule heating mechanism:

$$T_{ij} = T_0 + \sum_{j}^{N} \mathbf{K}_{ij} R_{ij} \mathbf{I}_{ij}^2. \tag{S4}$$

$T_{ij}$ denotes the local temperature of the $i, j$th MRR, $T_0$ is the room temperature, $\mathbf{K}$ denotes the effective thermo-optic coefficient matrix (diagonal in the absence of thermal crosstalk), and $R_{ij}$ is the electrical heater resistance. Moreover, recent study [2] further reveals that the temperature offset is also dependent on the MRR self-heating effect:

$$T_{ij} = \sum_{j}^{N} \alpha_{ij} P_{\text{self},ij} + \sum_{j}^{N} \mathbf{K}_{ij} R_{ij} \mathbf{I}_{ij}^2 + \sum_{j}^{N_t} \gamma_{ij} P_{\text{other},j} + T_0, \tag{S5}$$

where $N_t$ is the number of other thermal sources, $\alpha_{ij}, \gamma_{ij}$ represent the thermal coupling coefficients associated with self-heating and the other thermal sources, respectively. The self-heating power $P_{\text{self},ij}$ can be approximated as

$$P_{\text{self},ij} = -\frac{aQP_{j,\text{opt}}}{2\pi\nu_j}, \tag{S6}$$

where $a$ is the absorbance per unit time, $Q, \nu_j$ represents the quality factor of the MRR and the $j$th optical frequency, respectively.

Therefore, a simplified LUT accounting for the fabrication process variations, ambient temperature, input optical power, and the self heating effect, can be represented as

$$\mathbf{I}_{ij} = \tilde{f}_{ij}(\mathbf{w}_{ij}, \sigma_{ij}, T, P_{\text{opt}}), \tag{S7}$$

where the function $\tilde{f}_{ij}$ is determined by Eqs. (S1–S6).

## 2 Experimental Setup with Photonic Packaging

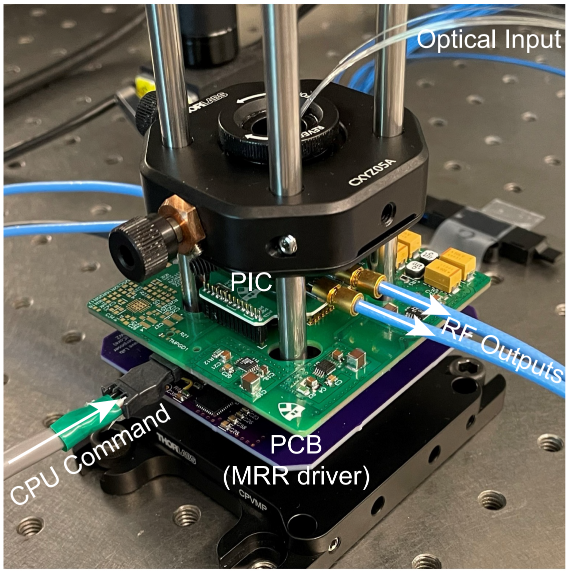


**Figure S1.** Photo of experimental setup. The photonic chip is co-packaged with the printed circuit boards (PCBs).

The experimental setup in this work shares a lot in common with the setup in ref. [3]. Similarly, a compact, portable experimental setup for the photonic integrated circuit (PIC) is shown in Fig. S1. Apart from the encapsulated PIC, the system requires three input channels, including three lasers (Pure-Photonics, PPCL500) and three Mach-Zehnder modulators (MZMs). The input light is coupled into the PIC by a vertical grating coupler with a roughly 6.5 dB coupling loss per facet. The output signals from the PIC are connected to voltage amplifiers (ZX60-153LN-S+, Mini-Circuits) via two coaxial cables, which are then collected by the scope and read by a peripheral CPU for further analysis. A grey ribbon cable sends digital commands from the CPU for programming the tuning currents and biasing voltages output by the digital-to-analogue converters (DACs, LTC2662 and LTC2664, Analog Devices) integrated onto the printed circuit board (PCB). The PCB is designed with electroless nickel immersion gold (ENIG) process pads connected to the PIC via wire bonding.

# 3 Demonstration on Iris Dataset

## 3.1 Definition of Loss function

In the proof-of-concept demonstration on Iris flower dataset, the task is converted from three-class classification to two-step binary classification. The conventional loss function for this two-step binary classification task is therefore defined as the sum of two binary cross-entropy loss functions:

$$\mathcal{L} = -\frac{1}{N}\sum_{i=1}^{N}\Big(1_{\{y_i=0\}}\log(p_1) + 0.5\cdot 1_{\{y_i=1\}}(\log(1-p_1)+\log(p_2)) + 0.5\cdot 1_{\{y_i=2\}}(\log(1-p_1)+\log(1-p_2))\Big),$$

where $N$ is the number of samples, $y_i(y_i = 0, 1, 2)$ denotes the label of Iris flowers and $p_{1,2}$ denotes the two predicted outputs. $1_{\{y_i=0,1,2\}}$ denotes the Kronecker delta function, which describes the condition for each label. According to Section 2.1.3 in the manuscript, the power-aware "pruned" loss function is defined as

$$\tilde{\mathcal{L}} = \mathcal{L} + \gamma\Sigma_{i,j}\mathbf{I}_{ij}^2 R, \tag{S8}$$

where $\gamma$ is the pruning strength, $\mathbf{I}_{ij}$ represents the tuning currents of six MRRs ($\mathbf{I}_{11}$, $\mathbf{I}_{12}$, $\mathbf{I}_{13}$, $\mathbf{I}_{21}$, $\mathbf{I}_{22}$, $\mathbf{I}_{23}$).

## 3.2 Data Generation and Modulation

It is crucial to ensure that the input data is accurately transmitted to the photonic chip. The three channels of input data (petal length, petal width, and sepal length) are converted to analog waveforms and sent to the Mach-Zehnder modulators by the arbitrary waveform generator (Keysight M8196A). To synchronize the output signals from the three different channels, we intentionally introduce circular time delays between the channels while generating the waveforms. Before sending the actual signals, we first define and send through testing waveforms of standard sinusoidal signals onto the three channels: $S = [S_1, S_2, S_3]$ where $S_2 = S_3 = -S_1$. The testing signals are sent through the photonic chip with no MRR weights applied and collected from the oscilloscope. Then, as shown in Fig. S2a, the output voltage amplitudes of the signal generator, as well as the time delays between $S_1$, $S_2$, and between $S_1$, $S_3$ are manually adjusted to ensure the sinusoidal signals from channel 1, 2 (and channel 1, 3) have the same amplitudes and the opposite optical phases. (Therefore, the testing signals on channel 1, 2 (or 1, 3) should perfectly cancel with each

other.) Additionally, there are external optical delay lines in the optical signal path to further ensure the optical phases in each path are aligned with each other.

To minimize intersymbol interference (ISI), the baseband signals for the Iris flower dataset are generated using the raised-cosine filter function with a sampling rate of 88 GSa/s (88 samples per symbol). The signals are then modulated onto optical carriers and processed by the PIC. Finally, the output data read by the oscilloscope are downsampled and further processed by the CPU. Fig. S2b shows the data collected by the scope after modulation and demodulation (no photonic weighting applied), which align with the original Iris features.

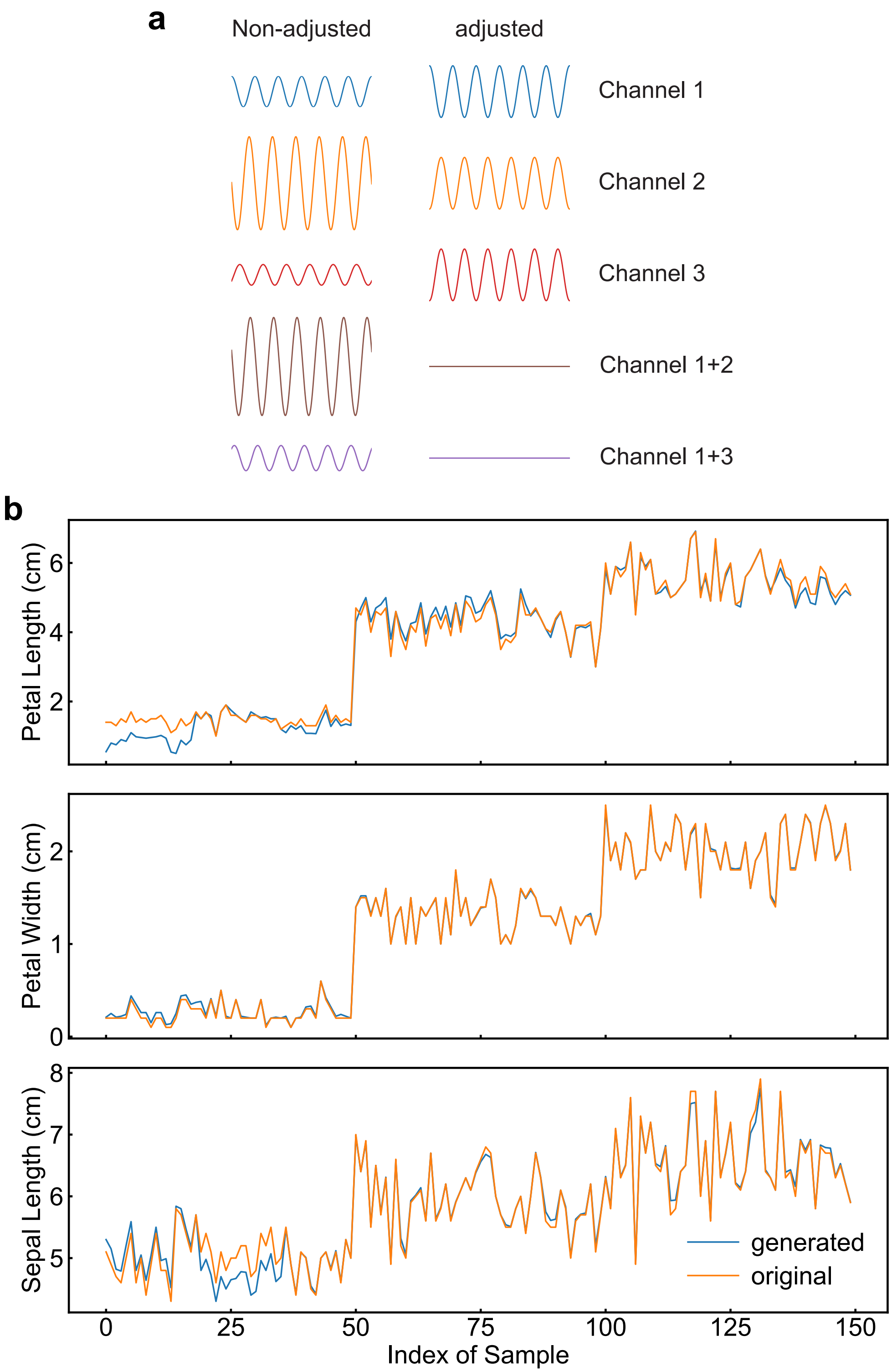


**Figure S2.** **a** Adjusting the test sinusoidal waveforms to synchronize three different channels. **b** Generated data of Iris flower dataset, ensuring that the input data is accurately transmitted to the PIC.

# 4 Simulations on Large-scale NNs

## 4.1 Datasets and NN setup

To investigate the scalability of our approach, simulations are performed on the widely used scikit-learn wine dataset, Modified National Institute of Standards and Technology (MNIST) dataset [4], the Canadian Institute for Advanced Research (CIFAR-10) dataset [5], and the Fashion MNIST dataset. The wine recognition dataset is a classic benchmark dataset that classifies three classes of wine based on 13 chemical attributes. The MNIST (Fashion-MNIST) dataset is a database of handwritten digits (fashion images), which has 60,000 28×28×1 grayscale training images, and 10,000 test images. The CIFAR-10 dataset consists of 60000 32×32×3 images (50000 for training and 10000 for testing) in 10 classes, with 6000 images per class.

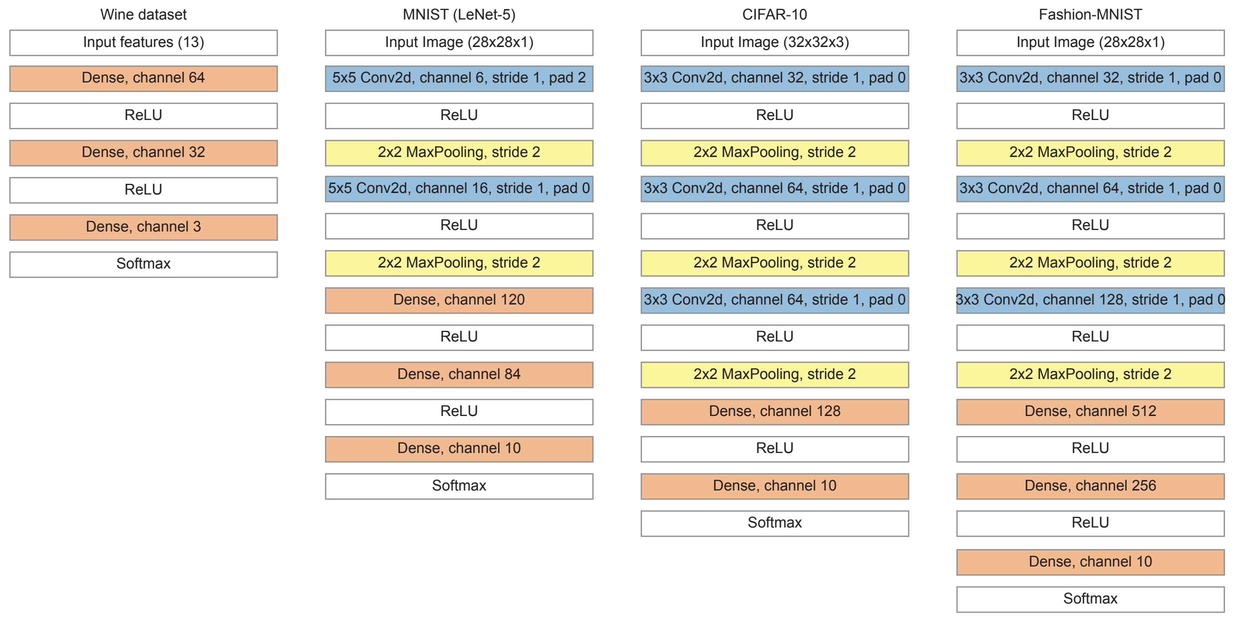


**Figure S3.** NN setup used in simulations on the wine, MNIST, CIFAR-10, and Fashion-MNIST datasets, respectively.

The detailed parameters of the NN setup used in the simulations are shown in Fig. S3. For the scikit-learn wine dataset, a three-layer fully-connected NN is used to categorize three classes of wine, which consists of 2,976 weight parameters. Furthermore, MRR-based convolutional neural networks (CNNs) are simulated for the image classification datasets, where 61,470, 188,512, 812,752 weights are needed for the MNIST, CIFAR-10, and Fashion-MNIST datasets, respectively.

## 4.2 Modeling and Procedure

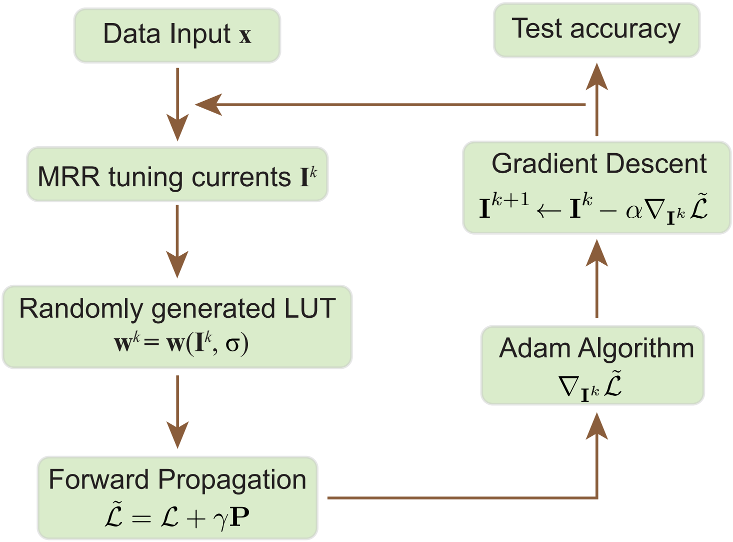


**Figure S4.** Procedure of online training and pruning simulations

According to Eqs. (S1–S5), the LUT can be effectively written as

$$\mathbf{w}_{ij}(\mathbf{I}_{ij}) = 1 - 2(1 + (\mathbf{I}_{ij}^2 - \mathbf{I}_{ij,0}^2)^2/\Delta\mathbf{I}_{ij}^2)^{-1}, \tag{S9}$$

where $\mathbf{w}_{ij}$ is the normalized MRR weight (between –1 and +1), $\mathbf{I}_{ij,0}$ is the offset determined by the MRR resonance wavelength offset $\sigma_{ij}$, $\Delta\mathbf{I}_{ij}$ is the half-width half maximum in the current unit. To simulate the MRR functional variations caused by fabrication and environmental variations, we induce random Gaussian shifts to the offset $\mathbf{I}_{ij,0}$. These variations are incorporated into our NN model, such that each MRR weight is calculated based on a differently generated LUT.

Fig. S4 shows the procedure of online training and pruning simulations. It follows the conventional tutorials of open-source software tools (such as PyTorch or Tensorflow), which include loading the dataset, building an NN model, and training and evaluating the model. As discussed in Section 2.1.2 of the manuscript, the trainable NN parameters are mapped from weights to MRR tuning currents using randomly generated LUTs considering the resonance variations.

### 4.3 Energy Saving Details via Pruning

As discussed in Section 2.1.3 and Fig. 5 of the manuscript, the trade-off between the testing accuracy and the overall energy savings via pruning for large-scale NNs is simulated. The entire simulated parameter space for each dataset is shown in Fig. S5 **a, c, e, g, i**. Fig. S5 **b, d, f, h, j** show the histograms of distributions of simulated MRR tuning power across five different datasets. For datasets using fully-connected NNs (Wine and Moons), (Fig. S5 **b, d**) that most of the MRR tuning powers can be reduced to almost zero with our pruning method (15% accuracy drop). In contrast, for larger datasets using CNNs (MNIST, CIFAR-10, Fashion-MNIST), as shown in Fig. S5 **f, h, j**, a fairly large amount of tuning power is still needed for some MRR weights to maintain the classification efficiency of CNNs, even with 15% error tolerance. These detailed results validate the scalability of our method, showing orders-of-magnitude reductions in power consumption if one can tolerate an error of 10% – 15%.

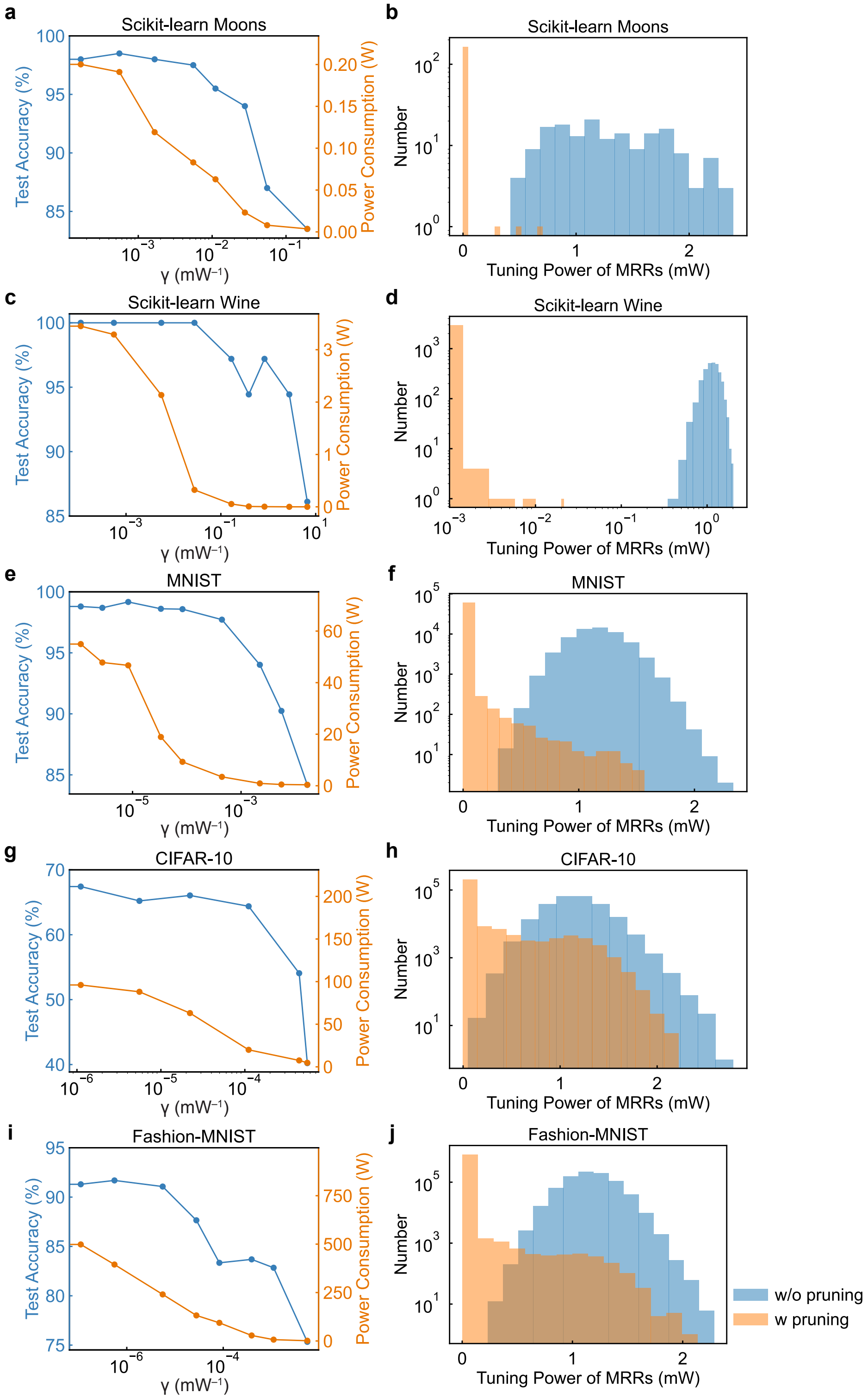


**Figure S5.** Simulation results of the energy savings resulting from pruning. **a** The tradeoff between the prediction accuracy and power consumption on Scikit-learn Moons dataset. **b** Histogram of distributions of MRR tuning power on Scikit-learn Moons dataset. **c, d** on Scikit-learn wine dataset. **e, f** on MNIST dataset. **g, h** on CIFAR-10 dataset. **i, j** on Fashion-MNIST dataset, respectively.

# 5 System Latency Analysis

Here, we present a detailed breakdown analysis of the system latency for each step in the online training and pruning experiment on Iris dataset. All the processing latencies are average of 1,000 repeated measurements. As summarized in Table S1, the actual time required for training each epoch includes the weight actuation time (35.3 ms for setting a single MRR weight, including the communication time between CPU and PIC driver), the on-chip optical inference time (5.3 ps, time-of-flight light propagation time), waveform downloading time from the oscilloscope (0.726 s for output from one MRR weight bank), and the time it takes to execute analog-to-digital conversions and calculations on CPU. Therefore, the total execution time of running one training epoch for Iris experiment is approximately 13.1 s, including additional latencies from Python code execution.

We also present an estimation of how the processing time for each step scales with the network size (e.g., on an MRR weight bank with an arbitrary size of $M \times N$). For the weight actuation time, it requires $MN$ actuations for each perturbation run and $MN + 1$ perturbations for each training epoch, so the estimated scaling factor is $O((MN)^2)$. For the on-chip optical inference time, the light propagation latency depends on the total length of the optical waveguides, which scales linearly with $(M + N)$. The scaling factor of scope waveform downloading time and analog-to-digital conversion time comes from the multiply of the total number of input samples $k$, the number of optical neurons (MRR weight banks) $M$, and the number of perturbations $(MN + 1)$. Therefore, the overall latency of our PNN setup approximately scales with $O(kM^2N)$, dominated by the slowest scope waveform downloading time.

However, We envision that the system training latency can be further reduced to the millisecond level by speeding up communications between equipments (e.g., replacing the oscilloscope with high-speed ADCs), as well as digital signal processing pipeline (e.g., replacing single-thread CPU processing with high-parallelism FPGA processing). This has been proven to be feasible in our earlier work [3].

| Processing Stages | Average Time | Times per Epoch | Est. Scaling Factor* |
|---|---|---|---|
| Weight Actuation** | 35.3 ms | $6 \times 7 = 42$ | $O((MN)^2)$ |
| On-chip Optical Inference | 5.3 ps | 7 | $O(MN(M+N))$ |
| Scope Waveform Download | 0.726 s | $2 \times 7 = 14$ | $O(kM^2N)$ |
| Analog-to-Digital Conversion | 20 µs | $2 \times 7 = 14$ | $O(kM^2N)$ |
| Calculation of Loss Function | 20 µs | 7 | $O(kM^2N)$ |
| Calculation of Gradient | 16 µs | 1 | $O(MN)$ |
| Total latency | 13.1 s | 1 | $O(kM^2N)$ |

**Table S1.** Detailed processing time for each step in the online training experiment. * Estimated scaling factor refers to how the corresponding times per epoch scales with an $M \times N$ MRR weight bank. $k$ is the total number of samples for input training data. ** includes the time for actuating the embedded N-doped heaters of the MRR, and the round-trip communication time between the CPU and the micro-controller (Tiny2040) on PCB, and between the micro-controller and the DAC.

## 6 Comparison with other works

| Ref (Year) | Algorithm | Est. Training Latency | Adaptive Training[1] | Dataset (Accuracies) |
|---|---|---|---|---|
| [6] (2021) | Genetic Algorithm | 2.08 ms[2] | No | Iris (93.3%) |
| [7] (2022) | Back-prop (Simulation) | Not given | No | Iris (95%) |
| [8] (2023) | Back-prop | Not given | No | Circle; Moons (96%; 94%) |
| [9] (2024) | Fully forward mode | 64 ms | No | Iris (94.2%) |
| [10] (2024) | SPSA[3] | Not given | No | Vowel (92.5%) |
| [11] (2025) | Back-prop | Not given | 0.5°C temp. change | Iris 93.3% |
| **This work** | Perturbation | 13.1 s[4] | **Yes** | Iris (99.33%) |

**Table S2.** Comparisons with other recent works on online training on integrated photonic platforms. [1]Adaptive Training: Experimentally demonstrated resilience to external temperature changes and fabrication process variations. [2] only includes weight actuation and PD measurement time. [3] Simultaneous Perturbation Stochastic Approximation. [4] limited by single-thread CPU processing and the communication time between equipments.

Table S2 shows a comprehensive comparison table of our work with other recent representative works on online training of integrated PNNs, in terms of training algorithms, estimated training latencies, adaptive training, and prediction accuracy in a given dataset. Although the concept of online training has been proposed and investigated on integrated photonic platforms, the experimental demonstration of the resilience of the photonic chip to external temperature changes and fabrication process variations is still lacking. In this work, we demonstrate for the first time that the training of PNN chips can operate in a wide temperature range of 26–40°C and with unpredictable fabrication variations. Despite the limited latency, our study serves as a fundamental methodology to address chip-to-chip variations in PICs and represents a significant milestone in the development of large-scale, energy-efficient MRR-based integrated analog photonic processors.

| Mechanism | Max. Tuning Speed (Hz) | Energy Consumption per bit | Requires Post-fab? | Nonvolatility | Tuning Range |
|---|---|---|---|---|---|
| Thermal-optic (w/o pruning) [12] | 175 k [13] | 30 nJ [14] | No | No | Large |
| Germanium Ion Implantation [15] | Static | 0 | Yes | Yes | Large |
| Phase-change Materials [16] | 50 k [17] | $\approx$ 100 nJ[1] [18] | Yes | Yes | Large |
| Carrier Depletion (Reverse-Bias PN) | 67 G [19] | 6.3 fJ [20] | No | No | Limited |
| Thermal-optic (w pruning) **(This work)** | 175 k [13] | **0.88 pJ**[2] | No | No | Large |

**Table S3.** Comparisons of our pruning method with other low-power tuning technologies for compensating FPVs. [1] includes the amorphization and crystallization pulse energy. [2] calculated based on the experimental on-chip MRR tuning power (5.28 mW for six MRRs) and the signal transmitting speed of 1 GBaud/s.

In Table S3, we present another comparative analysis of our pruning method with different tuning mechanisms for compensating fabrication process variations used in integrated silicon photonics. As discussed in the manuscript, the traditional thermal-optic tuning method remains necessary in a wide range of scenarios due to its large tuning range, despite slow speed (up to 175 kHz [13]) and high energy consumption (30 nJ/bit [14]). Post-fabrication methods including Germanium ion implantation [15] and phase change materials [16, 17] have been demonstrated for nonvolatile large range tuning, but they are either thermally instable or require additional I/O. Furthermore, high-speed MRR modulators based on the free carrier dispersion effect (in reverse-biased PN) offer tuning speed up to 67 GHz [19] and low energy consumption [20], but their tuning range is inherently limited by the small index change correct sub-FSR scale fabrication variations only. In our pruning work, we reduce the energy consumption per bit to 0.88 pJ (given the signal transmitting speed of 1 GBaud/s) while maintaining the tuning speed and range of thermal-optic tuning.

## References


[1] Ferreira de Lima, T., Doris, E.A., Bilodeau, S., Zhang, W., Jha, A., Peng, H.-T., Blow, E.C., Huang, C., Tait, A.N., Shastri, B.J., *et al.*: Design automation of photonic resonator weights. Nanophotonics **11**(17), 3805–3822 (2022)

[2] Lederman, J.C., Bilodeau, S., Doris, E., Blow, E.C., Zhang, W., Jimoh, Y., Shastri, B.J., Prucnal, P.R.: Low-latency passive thermal desensitization of a silicon micro-ring resonator with self-heating. APL Photonics **9**(7) (2024)

[3] Zhang, W., Lederman, J.C., Ferreira de Lima, T., Zhang, J., Bilodeau, S., Hudson, L., Tait, A., Shastri, B.J., Prucnal, P.R.: A system-on-chip microwave photonic processor solves dynamic RF interference in real time with picosecond latency. Light: Science & Applications **13**(1), 14 (2024)

[4] LeCun, Y., Bottou, L., Bengio, Y., Haffner, P.: Gradient-based learning applied to document recognition. Proceedings of the IEEE **86**(11), 2278–2324 (1998)

[5] Krizhevsky, A., Hinton, G., et al.: Learning multiple layers of features from tiny images (2009)

[6] Zhang, H., Thompson, J., Gu, M., Jiang, X.D., Cai, H., Liu, P.Y., Shi, Y., Zhang, Y., Karim, M.F., Lo, G.Q., *et al.*: Efficient on-chip training of optical neural networks using genetic algorithm. ACS Photonics **8**(6), 1662–1672 (2021)

[7] Ohno, S., Tang, R., Toprasertpong, K., Takagi, S., Takenaka, M.: Si microring resonator crossbar array for on-chip inference and training of the optical neural network. ACS Photonics **9**(8), 2614–2622 (2022)

[8] Pai, S., Sun, Z., Hughes, T.W., Park, T., Bartlett, B., Williamson, I.A., Minkov, M., Milanizadeh, M., Abebe, N., Morichetti, F., *et al.*: Experimentally realized in situ backpropagation for deep learning in photonic neural networks. Science **380**(6643), 398–404 (2023)

[9] Xue, Z., Zhou, T., Xu, Z., Yu, S., Dai, Q., Fang, L.: Fully forward mode training for optical neural networks. Nature **632**(8024), 280–286 (2024)

[10] Bandyopadhyay, S., Sludds, A., Krastanov, S., Hamerly, R., Harris, N., Bunandar, D., Streshinsky, M., Hochberg, M., Englund, D.: Single-chip

photonic deep neural network with forward-only training. Nature Photonics **18**(12), 1335–1343 (2024)

[11] Zhao, B., Wu, B., Sun, S., Zhang, S., Gao, D., Zhou, H., Dong, J., Zhang, X.: In-situ trained microring-based neural networks for scalable and robust photonic computing. Laser & Photonics Reviews, 01576

[12] Huang, C., Bilodeau, S., Ferreira de Lima, T., Tait, A.N., Ma, P.Y., Blow, E.C., Jha, A., Peng, H.-T., Shastri, B.J., Prucnal, P.R.: Demonstration of scalable microring weight bank control for large-scale photonic integrated circuits. APL Photonics **5**(4) (2020)

[13] Jayatilleka, H., Shoman, H., Chrostowski, L., Shekhar, S.: Photoconductive heaters enable control of large-scale silicon photonic ring resonator circuits. Optica **6**(1), 84–91 (2019)

[14] Watts, M.R., Sun, J., DeRose, C., Trotter, D.C., Young, R.W., Nielson, G.N.: Adiabatic thermo-optic Mach–Zehnder switch. Optics Letters **38**(5), 733–735 (2013)

[15] Milosevic, M.M., Chen, X., Cao, W., Runge, A.F., Franz, Y., Littlejohns, C.G., Mailis, S., Peacock, A.C., Thomson, D.J., Reed, G.T.: Ion implantation in silicon for trimming the operating wavelength of ring resonators. IEEE Journal of Selected Topics in Quantum Electronics **24**(4), 1–7 (2018)

[16] Fang, Z., Chen, R., Zheng, J., Khan, A.I., Neilson, K.M., Geiger, S.J., Callahan, D.M., Moebius, M.G., Saxena, A., Chen, M.E., *et al.*: Ultra-low-energy programmable non-volatile silicon photonics based on phase-change materials with graphene heaters. Nature Nanotechnology **17**(8), 842–848 (2022)

[17] Yang, X., Lu, L., Li, Y., Wu, Y., Li, Z., Chen, J., Zhou, L.: Non-volatile optical switch element enabled by low-loss phase change material. Advanced Functional Materials **33**(42), 2304601 (2023)

[18] Fang, Z., Zheng, J., Saxena, A., Whitehead, J., Chen, Y., Majumdar, A.: Non-volatile reconfigurable integrated photonics enabled by broadband low-loss phase change material. Advanced Optical Materials **9**(9), 2002049 (2021)

[19] Chan, D.W., Wu, X., Zhang, Z., Lu, C., Lau, A.P.T., Tsang, H.K.: Ultra-wide free-spectral-range silicon microring modulator for high capacity WDM. Journal of Lightwave Technology **40**(24), 7848–7855 (2022)

[20] Yuan, Y., Peng, Y., Sorin, W.V., Cheung, S., Huang, Z., Liang, D., Fiorentino, M., Beausoleil, R.G.: A 5×200 Gbps microring modulator silicon chip empowered by two-segment Z-shape junctions. Nature Communications **15**(1), 918 (2024)
